# *In vitro* anti-*Leishmania* activity of 8-hydroxyquinoline and its synergistic effect with amphotericin B deoxycholate against *Leishmania martiniquensis*

**DOI:** 10.7717/peerj.12813

**Published:** 2022-01-18

**Authors:** Wetpisit Chanmol, Padet Siriyasatien, Nuchpicha Intakhan

**Affiliations:** 1 School of Allied Health Sciences, Walailak University, Nakhon Si Thammarat, Thailand; 2 Research Excellence Center for Innovation and Health Product, Walailak University, Nakhon Si Thammarat, Thailand; 3 Hematology and Transfusion Science Research Center (HTSRC), Walailak University, Nakhon Si Thammarat, Thailand; 4 Vector Biology and Vector Borne Disease Research Unit, Department of Parasitology, Faculty of Medicine, Chulalongkorn University, Bangkok, Thailand

**Keywords:** Leishmaniasis, Leishmania martiniquensis, Mundinia, 8-Hydroxyquinoline, Synergistic effect, Drug combination, Amphotericin B deoxycholate

## Abstract

*Leishmania* (*Mundinia*) *martiniquensis* is responsible for visceral leishmaniasis in patients with no known underlying immunodeficiency, and visceral or disseminated cutaneous leishmaniasis in HIV-infected patients. The available anti-*Leishmania* drugs for treatment have limitations such as high toxicity and variable efficacy. To improve the therapeutic index of anti-*Leishmania* drugs, the search for a new drug or a new natural compound in combination therapy instead of using monotherapy to reduce drug side effect and have high efficacy is required. In this study, anti-*Leishmania* activity of 8-hydroxyquinoline (8HQN) and its synergistic effect with amphotericin B (AmB) against *L. martiniquensis* were evaluated *in vitro* for the first time. These results showed that 8HQN presented anti-*Leishmania* activity against *L. martiniquensis* with IC_50_ 1.60 ± 0.28 and 1.56 ± 0.02 µg/mL for promastigotes and intracellular amastigotes, respectively. The selectivity index (SI) value of 8HQN was 79.84 for promastigotes and 82.40 for intracellular amastigotes, which highlight promising results for the use of 8HQN in the treatment of *L. martiniquensis*-infected host cells. Interestingly, four combinations of 8HQN and AmB provided synergistic effects for intracellular amastigotes and showed no toxic effects to host cells. These results provided information of using a combination therapy in treating this *Leishmania* species leads to further development of therapy and can be considered as an alternative treatment for leishmaniasis.

## Introduction

Leishmaniasis is an infectious disease caused by different species of the protozoan parasite of the genus *Leishmania*, which invades a host to grow inside macrophages (Laranjeira-Silva, Hamza & Pérez-Victoria, 2020). *Leishmania* parasites are transmitted to humans and mammals through the bite of the sandfly vector that carries the infective metacyclic promastigotes (Giraud et al., 2019). Depending on the species of parasite, patients infected with *Leishmania* spp. may present a broad spectrum of clinical manifestations, from cutaneous (CL) to visceral leishmaniasis (VL) and be a carrier of parasites for long period with or without symptoms (Ghorbani & Farhoudi, 2017).

Both CL and VL disease are endemic in 56 and 55 countries, respectively. An estimated 50,000 to 90,000 new cases of VL and 600,000 to 1 million new cases of CL occur worldwide annually (WHO, 2021).

Leishmaniasis has been reported as emerging disease in several countries. In Southeast Asia, most of cases are infected with *Leishmania martiniquensis*, responsible for VL in a patient with no known underlying immunodeficiency, and VL or disseminated cutaneous leishmaniasis (DCL) in a HIV patient (Chusri et al., 2012; Pothirat et al., 2014). *L. martiniquensis* is of particular interest because it is in a new subgenus *L*. (*Mundinia*) (Espinosa et al., 2018) and few studies on drug treatment are available.

The treatment of leishmaniasis mainly relies on chemotherapy. Antimonials, miltefosine, paromomycin and amphotericin B deoxycholate (AmB) are antileishmanial drugs that have been used historically to treat a disease. However, there have been reports of antimony resistance in several regions, especially in the Indian subcontinent (Dumetz et al., 2018). Thus, miltefosine, paromomycin and AmB are increasingly used (Ponte-Sucre et al., 2017).

Most cases of *L. martiniquensis* infections were treated with AmB, since it is an available drug in many countries (Leelayoova et al., 2017). Recently, Phumee et al. (2020) using the colorimetric prestoblue resazurin-based assay, demonstrated that IC_50_ of amphotericin B was twice increased for *L. martiniquensis* (Phumee et al., 2020). Some fluorinated rhodacyanine analogues have shown better values than AmB (IC_50_ = 40–85 nM) against the promastigote and axenic amastigote stages (Lasing et al., 2020).

AmB is an antifungal agent, that provides reliable and broad-spectrum therapeutic alternative and also for use in the treatment of leishmaniasis. The drug causes the formation of an aqueous pore in cell membrane of the parasite, with an excellent leishmanicidal activity. However, the use of this drug is accompanied by dose-limited toxicities, highly toxic side effects, infusion-related reactions, and nephrotoxicity (Sundar & Chatterjee, 2006; Hamill, 2013). Therefore, to improve the therapeutic index of AmB, the search for a new drug or a new natural compound in combination with AmB therapy instead of using AmB in monotherapy may reduce drug side effects and have high efficacy.

8-Hydroxyquinoline (8HQN) is a plant-derived or synthetic quinoline. The compound has been used in agriculture, textile, wood and paper industries as a preservative, since it has the metal property of being a fungicidal activity (Short et al., 2006). Previous studies found that 8HQN showed great interest in a broad range of biological activities, such as antioxidant (Chobot et al., 2018), anticancer (Xie, Cai & Peng, 2018), antibacterial (Odingo et al., 2019), antifungal (Pippi et al., 2017), antiviral (Lai, Sridhar Prasad & Padmanabhan, 2013) and antischistosomal (Allam, Eweas & Abuelsaad, 2013) activities. It has been shown to have anti-*Leishmania* activity against *L. tropica*, *L. major* and *L. infantum* (Dardari et al., 2004). The compound was also effective against the intracellular stage of *L. braziliensis*, *L. amazonensis* and *L. infantum* with low toxicity to primary cells (Costa Duarte et al., 2016). The 8HQN has not been tested on *L. martiniquensis*. Thus, the aims of this study were to determine the anti-*Leishmania* activity of 8HQN, followed by the investigation of the synergistic effect between 8HQN and AmB against *L. martiniquensis*. To the best of knowledge, the results provided information of using a combination therapy in treating *L. martiniquensis* infection that may be useful for future treatment.

## Materials and methods

### Compound and drug

8HQN was commercially purchased (Sigma-Aldrich, Saint Louis, MO, USA). A stock solution of 8HQN (7,500 µg/mL) was prepared in 50% (v/v) ethanol (C_2_H_5_OH) and stored at −20 °C. AmB (250 µg/mL) was purchased (Gibco, Grand Island, NY, USA). Various concentrations of 8HQN and AmB were prepared in the fresh medium and used immediately.

### Parasites

*Leishmania martiniquensis* (MHOM/2013/LSCM3) was obtained from the Department of Parasitology, Faculty of Medicine, Chulalongkorn University. Promastigotes were grown in Schneider’s insect medium (SIM), pH 6.8 (Sigma-Aldrich, Saint Louis, MO, USA), containing 10% (v/v) heat-inactivated fetal bovine serum (FBS) (Gibco, Grand Island, NY, USA) and 25 µg/mL gentamicin sulfate (Sigma-Aldrich, Saint Louis, MO, USA) and incubated at 26 °C. Promastigotes were maintained in a logarithmic phase of growth by sub-passage every 3–4 days, starting the cultures with 10^6^ parasites/mL.

### THP-1 cells

The human monocytic cell line (THP-1) was used as a mammalian host cell for cytotoxicity assay, intracellular amastigote assay and drug combination assay. THP-1 cells were offered by Dr. Sirida Yangshim, Department of Microbiology, Faculty of Medicine, Chiang Mai University. THP-1 cells were cultured in RPMI-1640 medium (Gibco, Grand Island, NY, USA) supplemented with 10% FBS, 25 µg/mL gentamicin sulfate at 37 °C, 5%CO_2_. The sub-passaged was performed every 2–3 days, starting cultures with 5 × 10^4^ cells/mL. THP-1 cells were treated with 10 ng/mL of phobalmyristate acetate (PMA) (Sigma-Aldrich, Saint Louis, MO, USA) to induce macrophages differentiation before starting the experiment.

### Promastigote assay

To determine the anti-*Leishmania* activity of 8HQN against *L. martiniquensis* promastigotes, the logarithmic phase promastigotes were cultivated in the culture medium with the presence of various concentrations of 8HQN. AmB was used as a control. Parasite viability was assessed using alamarBlue cell viability assay (Thermo Fisher Scientific, Waltham, MA, USA) as described by Intakhan et al. (2020). Briefly, 8HQN (0.39–25 µg/mL) and AmB (0.004–0.625 µg/mL) were prepared in SIM supplemented with 10% FBS and 25 µg/mL gentamicin sulfate. A total of 50 µL of different dilutions of 8HQN and AmB were plated in a 96-well culture plate (Nunc, Roskilde, Denmark) and then added with 50 µL of 2 × 10^6^ logarithmic phase promastigotes of *L. martiniquensis*. Parasites were incubated with compound and drug at 26 °C for 48 h. To determine parasite proliferation using alamarBlue cell viability assay, 10 µL of alamarBlue reagent (Thermo Fisher Scientific, Waltham, MA, USA) was added into each well of a 96-well culture plate. The culture was further incubated for 24 h at 26 °C. Then, the absorbance was measured at wavelength of 570 and 600 nm using an Eon™ microplate spectrophotometer (BioTek, Winooski, VT, USA). Wells with medium alone and medium containing 10 µL of alamarBlue reagent were included as controls. Dose response curves obtained from % growth inhibition were generated using GraphPad Prism software (GraphPad Prism Software Inc., San Diego, CA, USA) The IC_50_ value (concentration of the compound or drug that inhibits 50% of the parasite viability after 72 h of incubation) was determined and expressed as mean ± SD. Results are from three independent experiments.

### Cytotoxicity assay

The cytotoxic activity of 8HQN was evaluated in THP-1 cells. Cells were treated with PMA to induce differentiation towards macrophages. Briefly, THP-1 cells were prepared in RPMI-1640 medium (10% FBS and 25 µg/mL gentamicin sulfate) containing 10 ng/mL of PMA. 100 µL of cells were placed into each well of 96-well culture plates (2.5 × 10^4^ cells/well). PMA-treated cells were incubated at 37 °C, 5%CO_2_ for 24 h to allow cell adhesion (complete differentiation). Then, cultures were washed and replaced with culture medium containing different dilutions of 8HQN (0.49–500 µg/mL), AmB (0.0049–10 µg/mL) and incubated for another 72 h at 37 °C, 5%CO_2_. To determine cell viability after exposure to compound and drug using alamarBlue cell viability assay, 10 µL of alamarBlue reagent was added into each well of a 96-well culture plate. Then, the incubation was continued for 4 h at 37 °C, 5%CO_2_ before reading the absorbance at wavelengths of 570 and 600 nm using an Eon™ microplate spectrophotometer. Wells with medium alone and medium containing 10 µL of alamarBlue reagent were included as controls. Dose response curves obtained from % survival of THP-1 cell were generated using GraphPad Prism software. The CC_50_ value (concentration of compound or drug that induces 50% of cell death after an incubation period of 72 h) was determined and expressed as mean ± SD. Results are from three independent experiments.

### Preparation of *Leishmania*-infected macrophages

Promastigotes were sub-passaged into RPMI-1640 medium supplemented with 20%FBS, 25 µg/mL gentamicin sulfate, pH 5.5 to stimulate metacyclogenesis (Zakai, Chance & Bates, 1998). Promastigotes were incubated at 26 °C for 5 days to yield metacyclic promastigotes. The culture with the presence of 70% of metacyclic promastigotes (body length < 12.5 µm, body width 1.5 µm and flagellum > body length) according to the criteria of Chanmol et al. (2019) was used for host cell infection. THP-1 cells were treated with 10 ng/mL of PMA for 24 h to differentiate into host cell macrophages in 96-well culture plates. Then, macrophages were infected with promastigotes (obtained from the culture with 70% metacyclic promastigotes) at a parasite/macrophage ratio of 10:1 to reach an optimal level of infection and incubated at 37 °C, 5%CO_2_.

### Intracellular amastigote assay

To determine the anti-*Leishmania* activity of 8HQN against *L. martiniquensis* amastigotes, intracellular amastigotes in THP-1 derived macrophages were treated with various concentrations of 8HQN. AmB was used as a control. The viability of amastigotes was assessed through the parasite rescue and transformation assay, a method described by Jain et al. (2012). Briefly, THP-1 cells were prepared in RPMI-1640 medium containing 10 ng/mL of PMA and dispended into 96-well culture plates. After 24 h of cell differentiation, THP-1 derived-macrophages were infected with promastigotes at a parasite/macrophage ratio of 10:1. Then, infected macrophages were treated with 8HQN (0.156–20 µg/mL) and AmB (0.004–0.5 µg/mL) prepared in RPMI-1640 medium supplemented with 2% FBS and 25 µg/mL gentamicin sulfate and incubated at 37 °C, 5%CO_2_. After exposure to the compound and drug for 48 h, survived amastigotes in macrophages were assessed by means of parasite rescue and formation assay. *Leishmania*-infected macrophages were washed with serum-free RPMI-1640 medium. Then, 0.05% (w/v) of sodium dodecyl sulfate (SDS) prepared in RPMI-1640 medium (20 µL/well) was added and incubated for 30 s to lyse the macrophage cell membrane and release intracellular amastigotes. Then, SIM supplemented with 20% FBS, 25 µg/mL gentamicin sulfate (180 µL/well) was added into each well to neutralize the SDS. The culture was incubated at 26 °C for 96 h to allow the amastigotes to grow, transform into promastigotes and start proliferation. Then, parasite proliferation was assessed using alamarBlue cell viability assay. The culture was added with alamarBlue reagent (20 µL/well) and incubated at 26 °C for another 24 h. Then, the absorbance was measured at wavelengths of 570 and 600 nm using an Eon™ microplate spectrophotometer. Wells with non-infected macrophage treated with 0.05% SDS (30 s) and medium containing 20 µL of alamarBlue reagent were included as controls. Dose-response curves were generated using GraphPad Prism software. The IC_50_ value was determined and expressed as mean ± SD. Results are from three independent experiments.

### Drug combination assay and analysis of interaction

**Figure 1:**
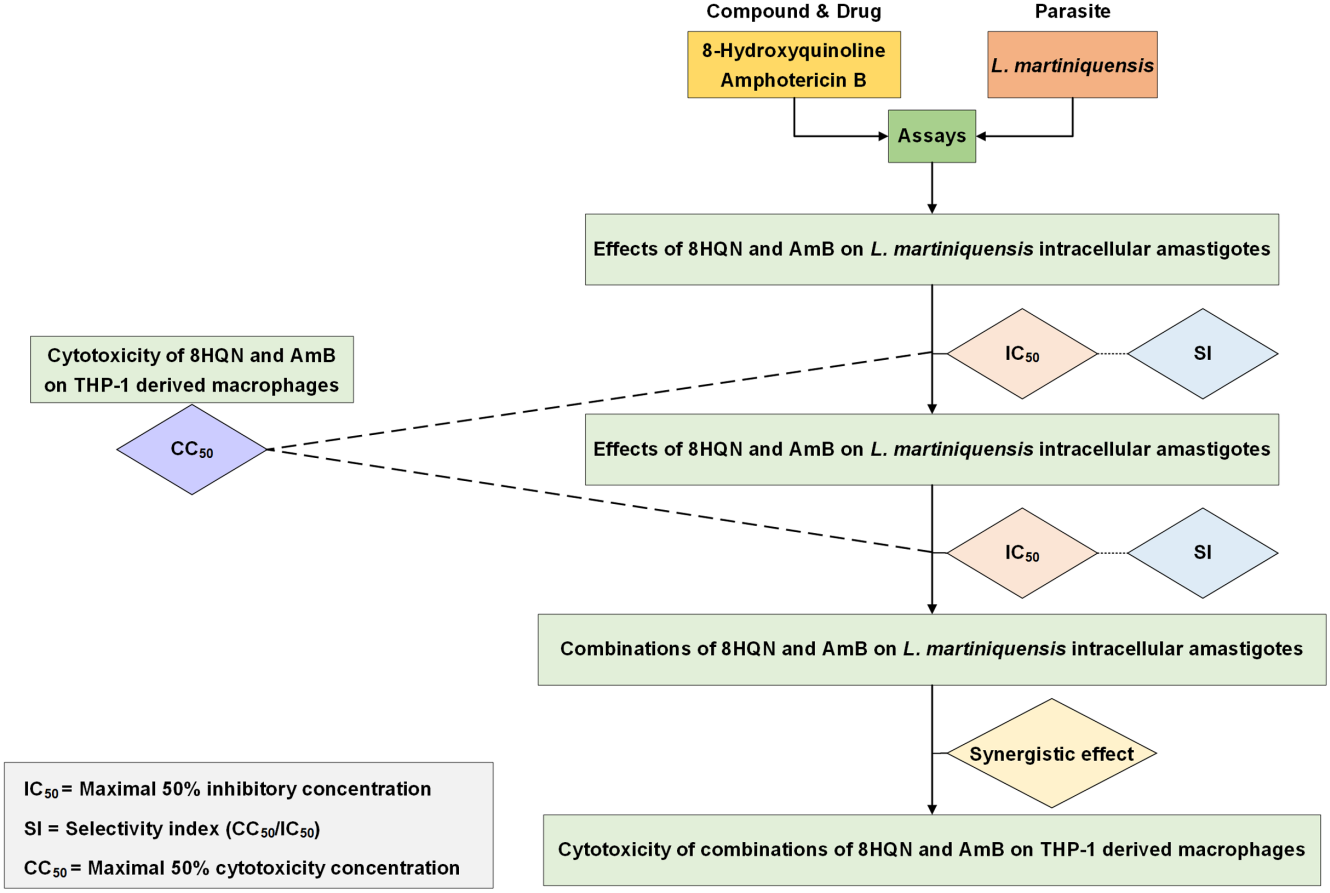
Overall study for 8HQN and AmB treatment in *L. martiniquensis*. The diagram shows processes for determining activity of 8HQN and AmB against promastigotes, intracellular amastigotes and THP-1 cells and processes for investigation of drug interaction and cytotoxicity of combinations.

Drug interaction was investigated by using the method as previously described by Intakhan et al. (2020). Double concentration of IC_50_ of 8HQN and AmB were prepared and serially diluted twice in 2%FBS RPMI-1640 medium. Different dilutions of 8HQN and AmB were combined using checker board assay (Zhao, Kelnar & Bader, 2014). The matrix of 8HQN/AmB provided 12 different combinations. *Leishmania*-infected macrophages were prepared in 96-well culture plates as describe above and treated with all combinations at 37 °C, 5%CO_2_ for 48 h. After exposure to combinations, macrophages surviving amastigotes were assessed using parasite rescue and transformation assay as described above. Promastigote proliferation in the culture at 96 h of culture was determined using alamarBlue cell viability assay. Each well was added with 20 µL of alamarBlue reagent and incubated for 24 h. Controls were wells with non-infected macrophages treated with 0.05% SDS (30 s) and medium containing 10 µL of alamarBlue reagent. Then, the absorbance was measured at wavelengths of 570 and 600 nm using an Eon™ microplate spectrophotometer (BioTek, Winooski, VT, USA). %Growth inhibition was used in the analysis of drug interaction. The interaction of 8HQN and AmB was analyzed using the Chou-Talalay combination index method by using CompuSyn software (Chou, 2008). Then, combinations were classified as synergism, additive or antagonism. Overall study is shown in Fig. 1.

### Statistical analysis

All data were recorded, edited and entered using GraphPad Prism software (GraphPad Prism Software, Inc., San Diego, CA, USA). Mean and standard deviation was calculated from three independent experiments. The statistical differences of %growth inhibition between drug combination and 8HQN alone was evaluated using one-way analysis of variance (ANOVA), followed by Dunnett’s multiple comparison tests using GraphPad Prism software (GraphPad Prism Software, Inc., San Diego, CA, USA). The statistical differences of cytotoxicity of drug combinations and drug or compound alone were evaluated using ANOVA, followed by Bonferroni’s multiple comparison tests. Differences were considered significant when *p* values were < 0.05.

## Results

### Effect of 8HQN against *L. martiniquensis* promastigote and intracellular amastigote

**Figure 2:**
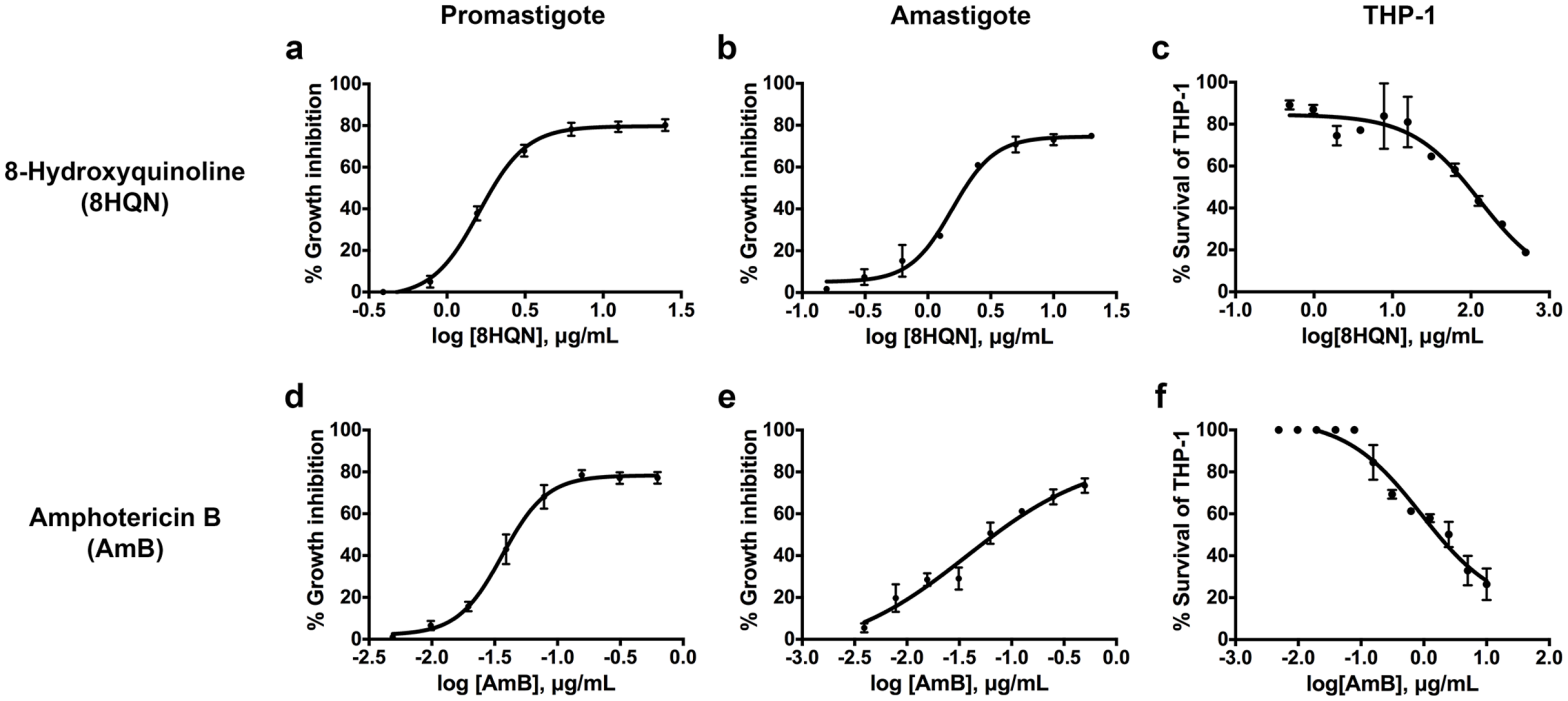
Dose response curves of 8HQN and AmB on *L. martiniquensis* and THP-1 cells. Parasites and THP-1 cells were treated with 8HQN and AmB. Percentages of parasite growth inhibition and THP-1 cell viability were determined using alamarBlue assay. Non-linear regression trendlines obtained by a serial compound or drug dilution were generated by using GraphPad Prism software. (A–C) Dose response curves of 8HQN for promastigotes, amastigotes and THP-1 cells, respectively. (D–F) Dose response curves of AmB for promastigotes, amastigotes and THP-1 cells, respectively.

8HQN has effect on both the promastigote and intracellular amastigote stages of *L. martiniquensis*. After 8HQN or AmB treatment, parasite growth was determined by the alamarBlue assay. The percentage of growth inhibition was plotted as a function of compound or drug concentration by fitting the values to a non-linear regression analysis. The dose response curves of 8HQN or AmB for promastigotes and amastigotes were generated by GraphPad Prism as shown in Fig. 2. These dose-response curves agree with the law of mass action and inhibition followed a sigmoidal curve. The IC_50_ value of 8HQN for promastigotes was 1.61 ± 0.28 µg/mL (Table 1). Intracellular amastigotes were more sensitive to 8HQN than promastigotes with IC_50_ value of 1.56 ± 0.02 µg/mL. The drug AmB showed IC_50_ value of 0.04 ± 0.02 µg/mL and 0.04 ± 0.01 µg/mL for promastigotes and amastigotes, respectively.

**Table 1:** Anti-*Leishmania* activity, cytotoxicity, and selectivity index of 8HQN and AmB. Growth of parasites and viability of THP-1 cells were determined using alamarBlue assay. The results were expressed as mean ± SD. The selectivity index (SI) was calculated by the ratio between the CC_50_ and IC_50_ values, respectively.

Biological activity			
Compound (µg/mL)	Promastigotes	Amastigotes	THP-1 cells
	IC_50_^a^/ SI^d^	IC_50_^b^/SI^d^	CC_50_^c^
8HQN	1.61 ± 0.28/79.84	1.56 ± 0.02/82.40	128.55 ± 0.92
AmB	0.04 ± 0.02/23.75	0.04 ± 0.01/23.75	0.95 ± 0.32

**Notes:**

a Inhibitory concentration for 50% of promastigotes.

b Inhibitory concentration for 50% of amastigotes.

c Cytotoxic concentration for 50% of THP-1 cells.

d Selectivity index.

### Cytotoxicity and selectivity index of 8HQN

The cytotoxicity of 8HQN was evaluated in THP-1 cells. The CC_50_ value of 8HQN was 128.55 ± 0.92 µg/mL, showing that it was less toxic to THP-1 cells than the drug, as shown in Table 1. The CC_50_ value of AmB was 0.95 ± 0.32 µg/mL. To compare the selectivity index (SI) of 8HQN and AmB, the SI value was calculated from the CC_50_ value divided by IC_50_ value. The SI values of 8HQN obtained from promastigotes and intracellular amastigotes were 79.84 and 82.40, respectively. The SI value of AmB was 23.75 for both promastigotes and intracellular amastigotes. These results showed that 8HQN was more effective in inhibiting parasite growth and safer to host cells than the drug.

### Synergistic effects of 8HQN in combination with AmB on *L. martiniquensis* amastigote in THP-1 cells

**Figure 3:**
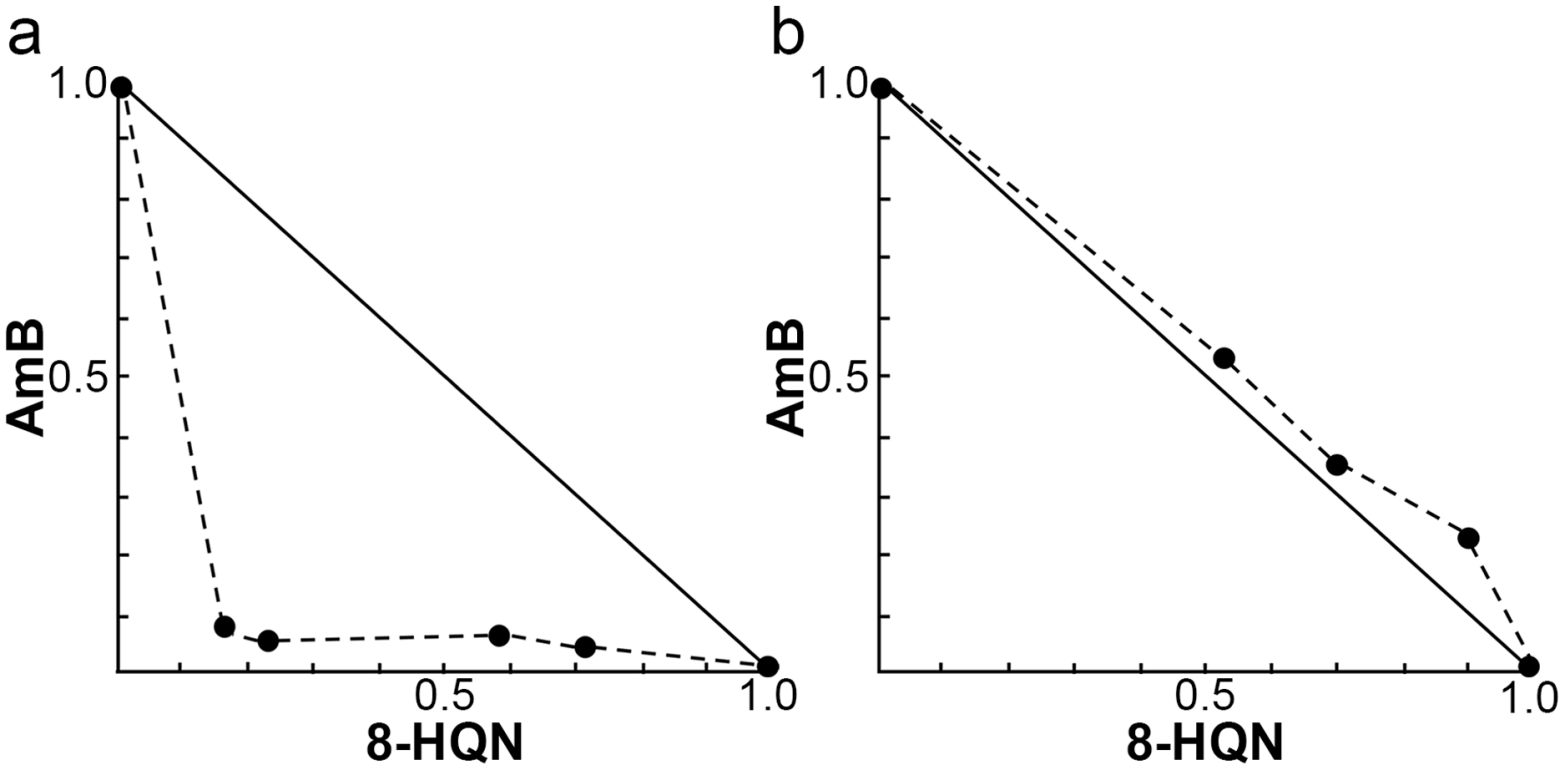
Representative normalized isobolograms of interaction between 8HQN and AmB against intracellular amastigote stage of *L. martiniquensis*. (A) 1.6 µg/mL 8HQN plus 0.05, 0.025, 0.0125 or 0.00625 µg/mL AmB. (B) 0.8 µg/mL 8HQN plus 0.05 or 0.025 µg/mL AmB. Data points (dots) located on, below, or above the line indicate additivity, synergy or antagonism, respectively.

The investigation of the synergistic effect of 8HQN in combination with AmB was performed using the Chou-Talalay combination index method. The IC_50_ values of 8HQN and AmB were 1.47 ± 0.02 µg/mL and 0.09 ± 0.02 µg/mL, respectively, which were similar to the previous IC_50_ results. The different concentrations of the combination were obtained from the checkerboard method. After treatment with each combination, the percentage of parasite growth inhibition was calculated from the percentage of treated parasites compared to untreated parasites (control) and then determined the CI value to classify the type of interaction, such as very strong synergism, strong synergism, synergism, moderate synergism, slight synergism, nearly additive, slight antagonism, moderate antagonism, antagonism, strong antagonism, or very strong antagonism. Results showed that the interaction between 8HQN and AmB was found from strong antagonism, nearly additive, to strong synergism as shown in Table 2. Two combinations of 8HQN/AmB (1.6:0.05 and 1.6:0.025 µg/mL) were classified as strong synergism. Combination of 1.6 µg/mL of 8HQN plus 0.0125 or 0.00625 µg/mL of AmB were classified as synergism and moderate synergism, respectively. Result showed nearly additive obtained from two combinations of 8HQN/AmB (0.8:0.05 and 0.8:0.025 µg/mL). The synergistic and additive effects of the combinations are presented as shown in the isobologram (Fig. 3). The result showed that four data points were below the line of additivity, indicating the synergy of 8HQN and AmB as shown in Fig. 3A. Some combinations indicated additive and antagonistic effects, showing data points near or above the line of additivity, as shown in Fig. 3B. A combination of 8HQN/AmB (1.6:0.00625 µg/mL) had the highest dose reduction index (DRI) values in the reduction of AmB used with DRI value of 22.7 (Table 2).

**Table 2:** Combinations of 8HQN and AmB on the intracellular amastigotes of *L. martiniquensis* in THP-1 cells.

Drug combination non-constant ratio (μg/mL)		%Growth Inhibition	Combination Index (CI)	Interpretation	Dose reduction index (DRI)	
8HQN	AmB				8HQN	AmB
0	0	0				
0.4	0	18.23 ± 5.41				
0.8	0	15.03 ± 2.62				
1.6	0	49.67 ± 4.16				
0	0.00625	16.43 ± 2.23				
0	0.0125	24.57 ± 1.37				
0	0.025	26.44 ± 1.80				
0	0.05	43.66 ± 1.34				
0.4	0.00625	1.00 ± 1.03***	4.10	Strong antagonism	0.60	0.16
0.4	0.0125	22.00 ± 3.58	1.80	Antagonism	1.84	3.80
0.4	0.025	20.00 ± 1.73	1.14	Slight antagonism	1.73	1.78
0.4	0.05	25.00 ± 0.96	1.45	Antagonism	2.02	1.04
0.8	0.00625	16.00 ± 3.98	1.51	Antagonism	0.74	6.15
0.8	0.0125	28.00 ± 4.53*	1.13	Slight antagonism	1.10	4.51
0.8	0.025	38.30 ± 1.85***	1.05	Nearly additive	1.42	2.90
0.8	0.05	51.00 ± 5.41****	1.05	Nearly additive	1.87	1.91
1.6	0.00625	68.50 ± 3.35**	0.76	Moderate synergism	1.40	22.70
1.6	0.0125	76.00 ± 6.55***	0.65	Synergism	1.72	13.88
1.6	0.025	94.50 ± 5.04****	0.29	Strong synergism	4.31	17.19
1.6	0.05	97.00 ± 3.60****	0.24	Strong synergism	6.07	12.07

**Note:**

8HQN and AmB were combined at a non-constant ratio. Dose dependent effects were analyzed by CompuSyn software. CI value of each combination was classified as very strong synergism (CI < 0.1), strong synergism (CI = 0.1–0.3), synergism (CI = 0.3–0.7), moderate synergism (CI = 0.7–0.85), slight synergism (CI = 0.85–0.90), nearly additive (CI = 0.90–1.10), slight antagonism (CI = 1.10–1.20), moderate antagonism (CI = 1.20–1.45), antagonism (CI = 1.45–3.30), strong antagonism (CI = 3.30–10), very strong antagonism (CI > 10). DRI is the fold dose of dose reduction allowed in a combination for a given degree of effect as compared with the dose of each 8HQN or AmB alone. Statistical differences between the effects of AmB alone and in combination with 8HQN are indicated as follows: *p ≤ 0.05; **p ≤ 0.01; ***p ≤ 0.001; ****p ≤ 0.0001.

### Cytotoxicity of 8HQN/AmB combinations to THP-1 derived macrophages

**Figure 4:**
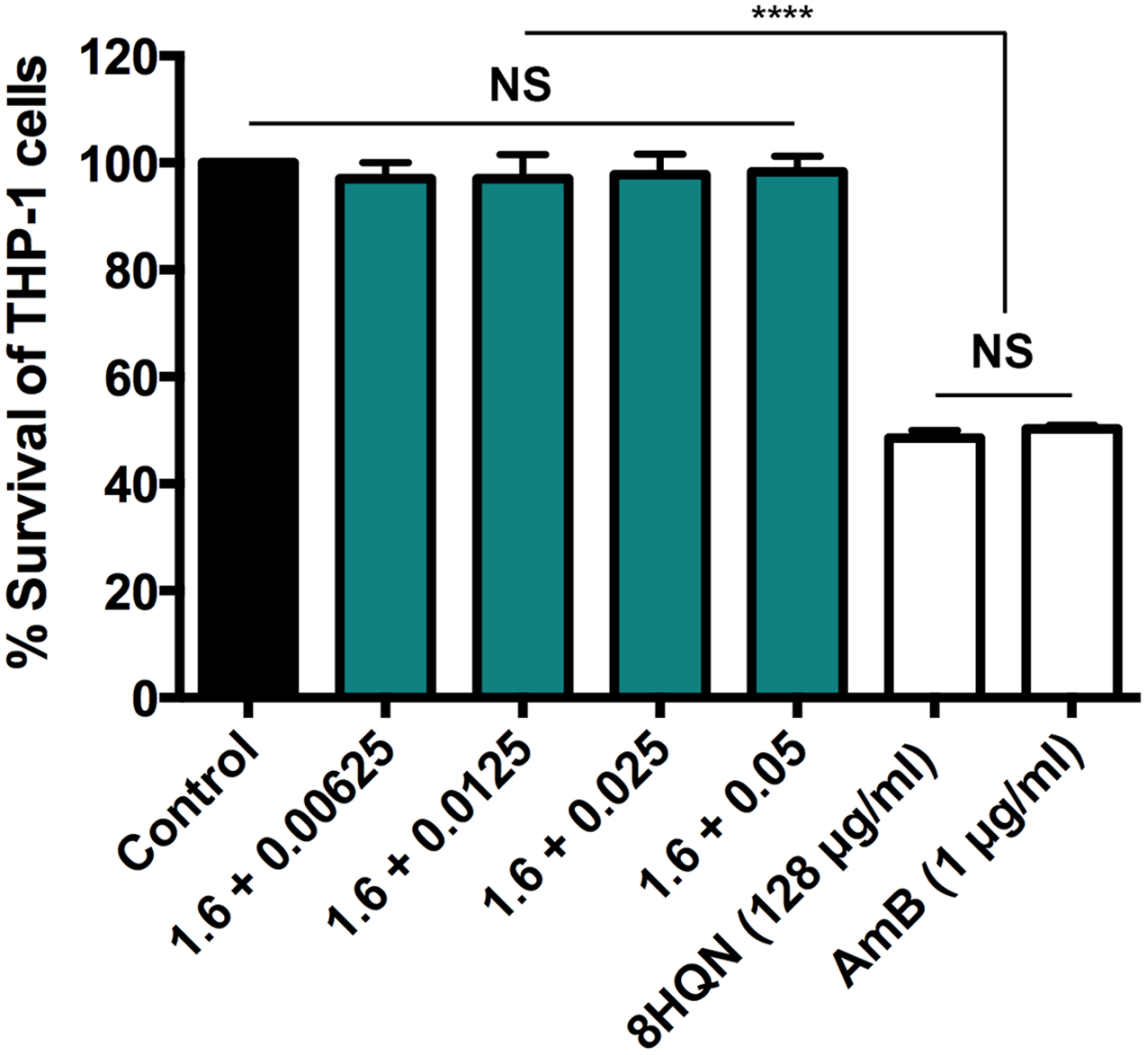
Cytotoxic activity of 8HQN in combination with AmB on THP-1 cells. The *x* axis represents different concentration (µg/mL) of 8HQN plus AmB. Solid bar, untreated control; Green bars, THP-1 cells treated with 1.6 µg/mL 8HQN plus 0.05, 0.025, 0.0125 or 0.00625 µg/mL AmB; white bars, THP-1 cells treated with 8HQN (128 µg/mL) or AmB (1 µg/mL). Statistical differences between the effects of 8HQN or AmB at CC_50_ values and their combinations (8HQN plus AmB) or untreated control are indicated as follows: NS, non-significance; ****, *p* ≤ 0.0001.

Four combinations (1.6 µg/mL 8HQN plus 0.05, 0.025, 0.0125 or 0.00625 µg/mL AmB) that provided synergistic effects (Table 2) were tested for their cytotoxicity to THP-1 derived macrophages. 8HQN and AmB, which were prepared at the concentration of CC_50_, were used as controls. The concentration of combinations providing synergistic effects against parasites showed no toxic effects for host cells, whereas both 8HQN and AmB at the CC_50_ concentration showed about 50% of the host cell viability, as shown in Fig. 4.

## Discussion

*L. martiniquensis* is a member of the subgenus *Leishmania* (*Mundinia*) and is one of the etiological agents of disseminated cutaneous and visceral leishmaniasis in humans (Chusri et al., 2012; Leelayoova et al., 2017; Espinosa et al., 2018; Burza, Croft & Boelaert, 2018; Valero & Uriarte, 2020). AmB is an available drug used for chemotherapy of *L. martiniquensis* infection (Leelayoova et al., 2017). It has proven to be effective against *L. martiniquensis in vitro* (Intakhan et al., 2020). However, the use of this drug is associated with highly toxic side effects (Grela et al., 2018). Thus, to improve the treatment and reduce the side effects of using monotherapy, the search for a new drug or a new natural compound in combination therapy that is less toxic and highly effective has been required.

In this study, the anti-*Leishmania* activity of 8HQN against *L. martiniquensis* was determined both in promastigote and intracellular amastigote stages of parasites, followed by the investigation of drug interaction between 8HQN and AmB. Previously, 8HQN has been shown to inhibit the multiplication of *L. tropica*, *L. major* and *L. infantum* at micromolar concentrations (Dardari et al., 2004). In the present study, *L. martiniquensis* was treated with 8HQN for the first time. 8HQN was able to inhibit the multiplication of promastigotes of *L. martiniquensis* in a microconcentration range. This confirms the efficacy of 8HQN, although this *Leishmania* species is in a distinct subgenus, *Leishmania* (*Mundinia*). The compound was also able to reach and inhibit the growth of intracellular amastigotes of *L. martiniquensis* within THP-1 derived macrophages, showing the ability to cross the host cell membrane and treat *Leishmania*-infected host cells. Interestingly, 8HQN had low toxicity to THP-1 derived macrophages, similar to a previous study in murine macrophages (Costa Duarte et al., 2016). Upon drug discovery, the compound with anti-*Leishmania* activity is expected to have less toxicity to host cells, showing an SI value higher than 10 (Don & Ioset, 2014).

Our results showed that 8HQN had SI values of 79.84 and 82.40 for promastigote and intracellular amastigote of *L. martiniquensis*, respectively. These SI values were higher than 10 and higher than the SI value of AmB, which highlight promising results for the use of 8HQN in the treatment of *Leishmania*-infected host cells. The mode of action of 8HQN is different, depending on the organism in which the compound acts. In *Schizosaccharomyces pombe* and *Clostridium difficile*, 8HQN acts as a chelating agent, which was able to inhibit RNA synthesis (Fraser & Creanor, 1974; Novakova et al., 2014). In intracellular microbes such as *Cryptococcus neoformans* and *Mycobacterium tuberculosis*, 8HQN activated the Cu-independent antibacterial host responses to kill intracellular pathogens (Festa et al., 2014; Shah et al., 2016). In the present study, a decrease in the number of intracellular amastigotes of *L. martiniquensis* in macrophages after treatment with 8HQN was similar to the results of a previous study in *L. braziliensis*, *L. amazonensis* and *L. infantum* (Costa Duarte et al., 2016). The compound failed to stimulate the production of nitric oxide by macrophages, showing that 8HQN did not activate the host response to kill parasites. However, some anti-*Leishmania* compounds showed the ability to kill parasites by up-regulating the level of ROS, causing oxidative stress (Mesquita et al., 2013). Costa Duarte et al. (2016) proved that 8HQN was unable to induce oxidative stress, but acted directly on parasite mitochondria, affecting the parasite mitochondrial membrane potential, causing parasite death. In addition, 8HQN did not affect the plasma membrane permeability of parasites, which is in contrast to the mechanism of AmB that acts on parasite plasma membrane by binding to ergosterols to form pores, resulting in parasite cell lysis (Mishra, Saxena & Singh, 2007). Furthermore, 8HQN alone and in a form of compound-containing polymeric micelle system have been proved to be effective against *L. amazonensis*-chronically infected BALB/c mice, suggesting the use of this compound *in vivo* (Costa Duarte et al., 2016; Lage et al., 2016).

In an attempt to reduce the side effects of AmB in monotherapy, 8HQN was combined with the drug and tested in intracellular amastigotes of *L. martiniquensis* to investigate its efficacy. Our results showed that 8HQN/AmB combinations provided synergistic effects without cytotoxicity to host cells. The combination between 8HQN 1.6 µg/mL and AmB 0.00625 µg/mL provided the highest DRI, showing approximately a 22-fold reduction in AmB used in the combination.

To date, there are no reports of synergistic effects of 8HQN in combination with AmB on any other species of *Leishmania*. Based on our finding, the synergistic effect of 8HQN with AmB on intracellular amastigotes of *L. martiniquensis*, can be explained by the ability of AmB to form pores in the parasite membrane, as well as potential direct effects of 8HQN on mitochondrial function.

Drug combinations were recommended to reduce the treatment duration and toxicity and delay the emergence of resistance (van Griensven et al., 2010). However, *Leishmania* parasites could develop resistance to drug combinations, leading to the potential risk of resistance to the different drugs used in combinations (García-Hernández et al., 2012). Thus, synergistic effects of 8HQN/AmB combinations found in this study can also be considered as an alternative combination therapy for leishmaniasis treatment.

## Conclusions

In conclusion, 8HQN and its combination with AmB were tested on *L. martiniquensis in vitro* for the first time. 8HQN was effective against *L. martiniquensis* and worked synergistically with AmB to reduce AmB used in the combination against parasites. These findings suggest that 8HQN can be considered as an alternative treatment for *L. martiniquensis* infection. The use of combinations may also bring significant advantages and better therapeutic effects than AmB alone.

## Supplemental Information

10.7717/peerj.12813/supp-1 Supplemental Information 1 Growth inhibition of promastigotes after exposure to 8HQN.

10.7717/peerj.12813/supp-2 Supplemental Information 2 Growth inhibition of promastigotes after exposure to AmB.

10.7717/peerj.12813/supp-3 Supplemental Information 3 Growth inhibition of intracellular amastigotes after exposure to 8HQN.

10.7717/peerj.12813/supp-4 Supplemental Information 4 Growth inhibition of intracellular amastigotes after exposure to AmB.

10.7717/peerj.12813/supp-5 Supplemental Information 5 Survival of THP-1 cells after exposure to 8HQN.

10.7717/peerj.12813/supp-6 Supplemental Information 6 Survival of THP-1 cells after exposure to AmB.

10.7717/peerj.12813/supp-7 Supplemental Information 7 Survival of THP-1 cells after exposure to combinations of 8HQN/AmB and 8HQN or AmB at CC50.

## Additional Information and Declarations

### Data Availability

The following information was supplied regarding data availability: The raw measurements are available in the Supplementary Files.

## References

[ref-1] Allam G, Eweas AF, Abuelsaad AS (2013). *In vivo* schistosomicidal activity of three novels 8-hydroxyquinoline derivatives against adult and immature worms of *Schistosoma mansoni*. Parasitology Research.

[ref-2] Burza S, Croft SL, Boelaert M (2018). Leishmaniasis. The Lancet.

[ref-3] Chanmol W, Jariyapan N, Somboon P, Bates MD, Bates PA (2019). Axenic amastigote cultivation and *in vitro* development of *Leishmania orientalis*. Parasitology Research.

[ref-4] Chobot V, Hadacek F, Bachmann G, Weckwerth W, Kubicova L (2018). Antioxidant properties and the formation of iron coordination complexes of 8-hydroxyquinoline. International Journal of Molecular Sciences.

[ref-5] Chou TC (2008). Preclinical versus clinical drug combination studies. Leukemia & Lymphoma.

[ref-6] Chusri S, Hortiwakul T, Silpapojakul K, Siriyasatien P (2012). Consecutive cutaneous and visceral leishmaniasis manifestations involving a novel *Leishmania* species in two HIV patients in Thailand. The American Journal of Tropical Medicine and Hygiene.

[ref-7] Costa Duarte M, dos Reis Lage LM, Lage DP, Mesquita JT, Salles BC, Lavorato SN, Menezes-Souza D, Roatt BM, Alves RJ, Tavares CA, Tempone AG, Coelho EA (2016). An effective *in vitro* and *in vivo* antileishmanial activity and mechanism of action of 8-hydroxyquinoline against *Leishmania* species causing visceral and tegumentary leishmaniasis. Veterinary Parasitology.

[ref-8] Dardari Z, Lemrani M, Bahloul A, Sebban A, Hassar M, Kitane S, Berrada M, Boudouma M (2004). Antileishmanial activity of a new 8-hydroxyquinoline derivative designed 7-[5′-(3′-phenylisoxazolino)methyl]-8-hydroxyquinoline: preliminary study. Farmaco.

[ref-9] Don R, Ioset JR (2014). Screening strategies to identify new chemical diversity for drug development to treat kinetoplastid infections. Parasitology.

[ref-10] Dumetz F, Cuypers B, Imamura H, Zander D, D’Haenens E, Maes I, Domagalska MA, Clos J, Dujardin JC, De Muylder G (2018). Molecular preadaptation to antimony resistance in *Leishmania donovani* on the Indian subcontinent. mSphere.

[ref-11] Espinosa OA, Serrano MG, Camargo EP, Teixeira M, Shaw JJ (2018). An appraisal of the taxonomy and nomenclature of trypanosomatids presently classified as *Leishmania* and *Endotrypanum*. Parasitology.

[ref-12] Festa RA, Helsel ME, Franz KJ, Thiele DJ (2014). Exploiting innate immune cell activation of a copper-dependent antimicrobial agent during infection. Chemistry & Biology.

[ref-13] Fraser RS, Creanor J (1974). Rapid and selective inhibition of RNA synthesis in yeast by 8-hydroxyquinoline. European Journal of Biochemistry.

[ref-14] García-Hernández R, Manzano JI, Castanys S, Gamarro F (2012). *Leishmania donovani* develops resistance to drug combinations. PLOS Neglected Tropical Diseases.

[ref-15] Giraud E, Martin O, Yakob L, Rogers M (2019). Quantifying *Leishmania* metacyclic promastigotes from individual sandfly bites reveals the efficiency of vector transmission. Communications Biology.

[ref-16] Ghorbani M, Farhoudi R (2017). Leishmaniasis in humans: drug or vaccine therapy?. Drug Design, Development and Therapy.

[ref-17] Grela E, Piet M, Luchowski R, Grudzinski W, Paduch R, Gruszecki WI (2018). Imaging of human cells exposed to an antifungal antibiotic amphotericin B reveals the mechanisms associated with the drug toxicity and cell defence. Scientific Reports.

[ref-18] Hamill RJ (2013). Amphotericin B formulations: a comparative review of efficacy and toxicity. Drugs.

[ref-19] Intakhan N, Chanmol W, Somboon P, Bates MD, Yardley V, Bates PA, Jariyapan N (2020). Antileishmanial activity and synergistic effects of amphotericin B deoxycholate with allicin and andrographolide against *Leishmania martiniquensis in vitro*. Pathogens.

[ref-20] Jain SK, Sahu R, Walker LA, Tekwani BL (2012). A parasite rescue and transformation assay for antileishmanial screening against intracellular *Leishmania donovani* amastigotes in THP1 human acute monocytic leukemia cell line. Journal of Visualized Experiments: JoVE.

[ref-21] Lage LM, Barichello JM, Lage DP, Mendonça DV, Carvalho AM, Rodrigues MR, Menezes-Souza D, Roatt BM, Alves RJ, Tavares CA, Coelho EA, Duarte MC (2016). An 8-hydroxyquinoline-containing polymeric micelle system is effective for the treatment of murine tegumentary leishmaniasis. Parasitology Research.

[ref-22] Lai H, Sridhar Prasad G, Padmanabhan R (2013). Characterization of 8-hydroxyquinoline derivatives containing aminobenzothiazole as inhibitors of dengue virus type 2 protease *in vitro*. Antiviral Research.

[ref-23] Laranjeira-Silva MF, Hamza I, Pérez-Victoria JM (2020). Iron and heme metabolism at the *Leishmania*-host interface. Trends in Parasitology.

[ref-24] Lasing T, Phumee A, Siriyasatien P, Chitchak K, Vanalabhpatana P, Mak KK, Hee Ng C, Vilaivan T, Khotavivattana T (2020). Synthesis and antileishmanial activity of fluorinated rhodacyanine analogues: the ‘fluorine-walk’ analysis. Bioorganic & Medicinal Chemistry.

[ref-25] Leelayoova S, Siripattanapipong S, Manomat J, Piyaraj P, Tan-Ariya P, Bualert L, Mungthin M (2017). Leishmaniasis in Thailand: a review of causative agents and situations. The American Journal of Tropical Medicine and Hygiene.

[ref-26] Mesquita JT, Pinto EG, Taniwaki NN, Galisteo AJ, Tempone AG (2013). Lethal action of the nitrothiazolyl-salicylamide derivative nitazoxanide via induction of oxidative stress in *Leishmania* (L.) *infantum*. Acta Tropica.

[ref-27] Mishra J, Saxena A, Singh S (2007). Chemotherapy of leishmaniasis: past, present and future. Current Medicinal Chemistry.

[ref-28] Novakova J, Džunková M, Musilova S, Vlkova E, Kokoska L, Moya A, D’Auria G (2014). Selective growth-inhibitory effect of 8-hydroxyquinoline towards *Clostridium difficile* and *Bifidobacterium longum* subsp. longum in co-culture analysed by flow cytometry. Journal of Medical Microbiology.

[ref-29] Odingo JO, Early JV, Smith J, Johnson J, Bailey MA, Files M, Guzman J, Ollinger J, Korkegian A, Kumar A, Ovechkina Y, Parish T (2019). 8-Hydroxyquinolines are bactericidal against *Mycobacterium tuberculosis*. Drug Development Research.

[ref-30] Pippi B, Reginatto P, Machado GDRM, Bergamo VZ, Lana DFD, Teixeira ML, Franco LL, Alves RJ, Andrade SF, Fuentefria AM (2017). Evaluation of 8-hydroxyquinoline derivatives as hits for antifungal drug design. Medical Mycology.

[ref-31] Ponte-Sucre A, Gamarro F, Dujardin JC, Barrett MP, López-Vélez R, García-Hernández R, Pountain AW, Mwenechanya R, Papadopoulou B (2017). Drug resistance and treatment failure in leishmaniasis: a 21st century challenge. PLOS Neglected Tropical Diseases.

[ref-32] Pothirat T, Tantiworawit A, Chaiwarith R, Jariyapan N, Wannasan A, Siriyasatien P, Supparatpinyo K, Bates MD, Kwakye-Nuako G, Bates PA (2014). First isolation of *Leishmania* from Northern Thailand: case report, identification as *Leishmania martiniquensis* and phylogenetic position within the *Leishmania enriettii* complex. PLOS Neglected Tropical Diseases.

[ref-33] Phumee A, Jariyapan N, Chusri S, Hortiwakul T, Mouri O, Gay F, Limpanasithikul W, Siriyasatien P (2020). Determination of anti-leishmanial drugs efficacy against *Leishmania martiniquensis* using a colorimetric assay. Parasite Epidemiology and Control.

[ref-34] Shah S, Dalecki AG, Malalasekera AP, Crawford CL, Michalek SM, Kutsch O, Sun J, Bossmann SH, Wolschendorf F (2016). 8-Hydroxyquinolines are boosting agents of copper-related toxicity in *Mycobacterium tuberculosis*. Antimicrobial Agents and Chemotherapy.

[ref-35] Short BR, Vargas MA, Thomas JC, O’Hanlon S, Enright MC (2006). *In vitro* activity of a novel compound, the metal ion chelating agent AQ+, against clinical isolates of *Staphylococcus aureus*. The Journal of Antimicrobial Chemotherapy.

[ref-36] Sundar S, Chatterjee M (2006). Visceral leishmaniasis – current therapeutic modalities. The Indian Journal of Medical Research.

[ref-37] Valero NNH, Uriarte M (2020). Environmental and socioeconomic risk factors associated with visceral and cutaneous leishmaniasis: a systematic review. Parasitology Research.

[ref-38] van Griensven J, Balasegaram M, Meheus F, Alvar J, Lynen L, Boelaert M (2010). Combination therapy for visceral leishmaniasis. The Lancet Infectious Diseases.

[ref-39] WHO (2021). Situation and trends of leishmaniasis. https://www.who.int/data/gho/data/themes/topics/topic-details/GHO/leishmaniasis.

[ref-40] Xie F, Cai H, Peng F (2018). Anti-prostate cancer activity of 8-hydroxyquinoline-2-carboxaldehyde-thiosemicarbazide copper complexes *in vivo* by bioluminescence imaging. JBIC Journal of Biological Inorganic Chemistry.

[ref-41] Zakai HA, Chance ML, Bates PA (1998). *In vitro* stimulation of metacyclogenesis in *Leishmania braziliensis*, *L. donovani*, *L. major* and *L. mexicana*. Parasitology.

[ref-42] Zhao J, Kelnar K, Bader AG (2014). In-depth analysis shows synergy between erlotinib and miR-34a. PLOS ONE.

